# The function of gut microbiota in immune-related neurological disorders: a review

**DOI:** 10.1186/s12974-022-02510-1

**Published:** 2022-06-15

**Authors:** Panida Sittipo, Jaeyoon Choi, Soojin Lee, Yun Kyung Lee

**Affiliations:** 1 Department of Integrated Biomedical Science, Soonchunhyang Institute of Medi-Bio Science, Soonchunhyang University, Cheonan, 31151 Republic of Korea; 2 Department of Microbiology and Molecular Biology, Chungnam National University, Daejeon, 34134 Republic of Korea

**Keywords:** Gut microbiota, Neurological disorders, Gastrointestinal tract, Microbiota–gut–brain axis

## Abstract

This review provides an overview of the importance of microbiota in the regulation of gut–brain communication in immune-related neurological disorders. The gastrointestinal (GI) tract hosts a diverse abundance of microbiota, referred to as gut microbiota. The gut microbiota plays a role in the maintenance of GI tract homeostasis and is likely to have multiple effects on brain development and function. The bidirectional communication between the gut microbiota and the brain is termed the microbiota–gut–brain axis. This communication between the intestine and the brain appears to affect human health and behavior, as certain animal studies have demonstrated the association between alterations in the gut microbiota and neurological disorders. Most insights about the microbiota–gut–brain axis come from germ-free animal models, which reveal the importance of gut microbiota in neural function. To date, many studies have observed the impact of the gut microbiota in patients with neurological disorders. Although many studies have investigated the microbiota–gut–brain axis, there are still limitations in translating this research to humans given the complexities of the relationship between the gut microbiota and the brain. In this review, we discuss emerging evidence of how the microbiota–gut–brain axis regulates brain development and function through biological networks, as well as the possible contribution of the microbiota–gut–brain axis in immune-related neurological disorders.

## Background

The gut microbiota residing in the gastrointestinal (GI) tract plays an important role in the health status of the host by regulating the cells in local and distant organs, including the brain. Recent studies have demonstrated that the gut microbiota plays a critical role in the regulation of brain function and host immunity [1–6]. The biological network of bidirectional communication between the gut microbiota and the brain is referred to as the “microbiota–gut–brain axis” [5, 7, 8]. A healthy gut microbiota benefits the host by producing microbial metabolites and neurotransmitters for communication with the host cells, such as intestinal epithelial cells (IECs) and immune cells. Alterations in the gut microbiota and microbial metabolite production have been linked to a wide range of immune-related neurological disorders, including developmental disorders, neurodegeneration, and emotional dysregulation. Furthermore, the gut microbiota plays a major role in the modulation of disease outcomes. This review aims to highlight the role of the gut microbiota and microbial metabolites in brain function and development, the proposed mechanisms underlying the communication between the gut microbiota and the brain, and the alterations in the gut microbiota in immune-related neurological disorders.

## Role of the gut microbiota in brain function and development

**Fig. 1:**
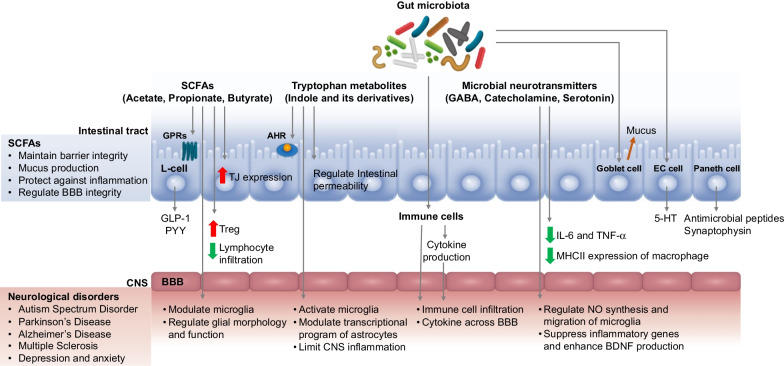
Summary of the mechanism by which the gut microbiota mediate the microbiota–gut–brain axis. The gut microbiota produce metabolites (SCFAs and tryptophan metabolites) and microbial neurotransmitters (GABA, catecholamine, and serotonin). The gut microbiota and their metabolites further impact IECs and the immune system, which mediate the pathology of neurological disorders

The GI tract is a highly complex organ composed of microorganisms, the intestinal epithelium, and the mucosal immune system. The microorganisms living in the GI tract, including bacteria, archaea, fungi, and viruses, are termed the gut microbiota [9, 10]. Human health can be both positively and negatively regulated by the gut microbiota. The gut microbiota benefit the host by converting dietary nutrients into microbial metabolites that communicate with one other and with the host cells, which further impacts host health and disease status [11, 12]. The gut microbiota and microbial metabolites not only play a role in the maintenance of gastrointestinal homeostasis, but also provide signals to distant organs in the body, including the brain [1–4]. Over the past decade, it has been known that the communication in the microbiota–gut–brain axis enables the gut microbiota to connect to immune and hormonal systems in the regulation of brain function and development [1–4]. The developmental parallels of the gut and brain during early life are known [13, 14]. The gut microbiota is diverse and rich during early life and its disruption at this critical period can affect brain development and function [15]. For example, infants with high levels of *Bacteroides* had better cognitive outcomes, while those who had high alpha diversity (the diversity of species within each individual) of gut microbiota showed lower scores on the overall composite score, visual reception scale, and expressive language scale [13]. Colonization of gut microbiota in early life plays an important role in the development and maturation of the immune and endocrine systems, both of which influence the central nervous system (CNS) function [2, 5, 6]. Studies on germ-free (GF) animals or broad-spectrum antibiotic-treated animals are commonly utilized to study the microbiota–gut–brain axis, particularly the impact of complete absence of the gut microbiota on development and behavior [2, 16, 17]. GF mice exhibit impaired brain function in learning, recognition, and behavior [16, 17]. In addition, the levels of important neurotransmitters, such as serotonin or 5-hydroxytryptamine (5-HT) and brain-derived neurotropic factor (BDNF), are altered compared to those in conventional mice [16, 18]. GF mice exposed to gut microbiota in early life demonstrate similar behaviors as specific pathogen-free mice, suggesting that early life is a sensitive period for the gut microbiota to regulate brain development and behavioral functions [16]. Microglia are major immune cells that maintain CNS processes and homeostasis [19]. Recent studies have highlighted the role of the gut microbiota in regulating microglial maturation and function [20]. GF mice and antibiotic-treated mice showed significant microglial defects with a reduction in the number of immature phenotypes and altered inflammatory cytokine profiles that influence the basal surveillance (M0) state [20]. In addition, the maturation of the CNS may be regulated by the gut microbiota [21]. The blood–brain barrier (BBB) is a selective semipermeable border of endothelial cells that acts as a gatekeeper to prevent harmful substances from entering the brain and ensures homeostasis of the CNS. GF mice displayed increased BBB permeability with reduced expression of tight junction (TJ) proteins [22], which may allow harmful molecules to enter the brain and cause neuroinflammation and damage. However, GF mice exposed to the gut microbiota from pathogen-free mice displayed increased integrity of the BBB [22], These studies highlight the role of the gut microbiota in regulating neuroimmunity and brain function. A summary of how the gut microbiota mediates the microbiota–gut–brain axis is shown in Fig. 1.

## Proposed mechanisms underlying communication between the gut microbiota and the brain

Recent studies have shown how microorganisms influence the brain through their ability to produce and modify many metabolic, immunological, and neurochemical factors in the gut that ultimately impact the CNS. In this section, we review the three proposed mechanisms that the gut microbiota use for communication with the nervous system.

### Bacterial metabolites/neurotransmitters

Microbial metabolites are important factors for communication among the gut microbiota and between the gut microbiota and host cells [11, 12]. Currently, many researchers are focusing on how the gut microbiota influences brain function and behaviors through their ability to produce microbial metabolites and neurotransmitters that can readily cross the BBB or otherwise activate other pathways [20]. The metabolites and neurotransmitters that are produced by the gut microbiota, and their functions, are listed in Table 1.

**Table 1:** Functions of the metabolites and neurotransmitters produced by the gut microbiota

Microbial metabolites/ neurotransmitters	Gut microbiota (Genus)	Functions	Neurological diseases
SCFAs	*Faecalibacterium*, *Clostridium*, *Eubacterium*, *Roseburia*, *Anaerostipes*, *Bifidobacterium*, *Akkermansia*	• Act as an energy source for colonocytes and regulate the intestinal barrier • Exert anti-inflammatory effects on the intestinal mucosal and CNS • Regulate GLP production which further influences neuronal inflammation • Regulate the integrity of the BBB	• Promote microglia activation and Aβ plaque deposition in AD • Accelerate α-syn aggregation and promote motor dysfunction in PD • Inhibit neuroinflammation and alleviate neurological damage in PD via improvement of motor impairment and dopaminergic neuron degeneration • Ameliorate EAE via induction of Treg, reduced lymphocyte infiltration, and demyelination
Tryptophan metabolites	*Lactobacillus*, *Escherichia*, *Clostridium*, *Bacteroides*, *Bacillus*, *Burkholderia*, *Streptomyces*, *Pseudomonas*	• Regulate microglial activation and the production of TGFα and VEGF-B • Impact neuronal proliferation, differentiation, and neurogenesis	• Indole decreases motor activity and enhances anxiety-like behavior • Kynurenine disrupts neurotransmission, leading to depression and altered brain function • Indole and IAA help in pathogen colonization in the gut, which negatively alters the function of the gut–brain axis in autism
GABA	*Lactobacillus*, *Bifidobacterium*, *Streptococcus*	• Down-regulate pro-inflammatory cytokine production and up-regulate immunoregulatory molecules • Modulate the inhibitory-excitatory balance necessary for brain function • Regulate the secretion of neuropeptides by intrinsic and extrinsic intestinal nerve fibers	• Ameliorate EAE via inhibition of inflammation, directly acting on APCs and adaptive immune cells in response to myelin proteins • Reduce stress-related disorders, such as anxiety and depression via changing cerebral GABAergic activity
Dopamine	*Bacillus*, *Staphylococcus*, *Proteus*, *Serratia*, *Escherichia*	• Modulate the function of effector immune cells and the production of cytokines by activated T cells • Reduce the suppressive activity and migratory activity of Treg • Regulate nitric oxide synthesis and migration of microglia	• Downregulate the production of IFN-γ and IL-17A by PBMCs in patients with relapsing–remitting MS
Norepinephrine	*Escherichia*, *Bacillus*, *Saccharomyces*, *Proteus*, *Serratia*	• Suppress inflammatory gene transcription and enhance BDNF production by microglia and astrocytes, which can further promote neuronal survival • Modulate excitatory neuronal responses and inter-neuronal responses	• Reduce Parkinson’s disease progression by inhibiting microglial activation and suppressing pro-inflammatory cytokine production
Serotonin	*Candida*, *Streptococcus*, *Escherichia*, *Enterococcus*, *Pseudomonas*, *Streptococcus*	• Suppress MHC class II expression and the antigen-presenting capacity of macrophages • Decrease the production of pro-inflammatory cytokines, such as IL-6 and TNF-α by macrophages and lymphocytes	• Attenuate MS or EAE by suppressing T cell proliferation, stimulating IL-10 production, inhibiting the release of IL-17A and IFN-γ, and inducing macrophage polarization into M2 macrophages

#### Short-chain fatty acids (SCFAs)

SCFAs are the main metabolites produced by the bacterial fermentation of dietary fibers. Acetate, propionate, and butyrate are the main SCFAs. In the human GI tract, the colon contains the highest SCFA concentration in a ratio of 60:20:20 for acetate, propionate, and butyrate, respectively [23]. The gut microbiota differ significantly in their potential to produce enzymes for SCFA formation. Bacteria in the phylum *Firmicutes* have been well-described as the predominant producers of butyrate [24]. In addition, other butyrate-producing gut microbiota belong to the genera *Faecalibacterium*, *Clostridium*, *Roseburia*, *Eubacterium*, and *Anaerostipes* [25, 26]. *Bifidobacterium* spp. produce acetate [27]. In addition, both acetate and propionate are formed from mucin fermentation by the mucin-degrading bacteria *Akkermansia muciniphila* [28]. Communication between the gut microbiota also supports the production of SCFAs, for example, *Bacteroides thetaiotaomicron* produces acetate, which is further utilized by *Eubacterium hallii* to generate butyrate [29]. SCFAs are absorbed by colonocytes, mainly via monocarboxylate transporters (MCTs), and serve as energy sources for colonocytes [30, 31]. In addition, SCFAs enhance the integrity of the intestinal epithelial barrier by facilitating the assembly of TJs [32]. Moreover, SCFAs also facilitate regulatory T cell (Treg) generation and homeostasis [33, 34]. In addition to exerting local effects in the intestinal tract and peripheral tissues, SCFAs play a pivotal role in microbiota–gut–brain crosstalk. SCFAs bind to G protein-coupled receptors (GPCRs), such as GPR41, GPR43, and GPR109a, which are expressed in various cell types [35]. The outcomes of receptor activation differ depending on the cell in which they are expressed. For example, SCFAs bind to their receptors on enteroendocrine cells and stimulate the secretion of glucagon-like peptide 1 (GLP-1) and peptide YY (PYY), which further regulates neuroinflammation [36]. GF mice monocolonized with a single bacterial strain that mainly produces SCFAs or treated with sodium butyrate showed decreased BBB permeability and increased expression of brain endothelial TJs compared to untreated GF mice [22]. It has been reported that the administration of sodium butyrate could prevent BBB breakdown and promote neurogenesis via inhibition of histone deacetylation [37, 38]. Enhanced BBB integrity by SCFAs plays a crucial role in controlling the passage of nutrients from the circulation to the brain and in maintaining CNS homeostasis. Propionate can interact with free fatty acid receptor-3 (FFAR3) on endothelial cells, resulting in the inhibition of non-specific microbial infections and protection of the BBB from oxidative stress [39]. The well-known transporters of SCFAs, MCTs, are abundantly expressed in endothelial cells and brain tissue [40, 41]. The crossing of the BBB by SCFAs is possibly facilitated by MCTs in endothelial cells [30, 41]. The presence of SCFAs in the human brain reflects their ability to cross the BBB [42]. SCFAs that cross into the CNS can be recognized by microglia, astrocytes, and neurons that sequentially modulate neurological and behavioral processes [43]. The gut microbiota-depleted mice demonstrated altered inflammatory gene expression profiles and immature states of microglia. However, this dysfunction can be restored by SCFAs [20]. In addition, SCFAs treatment could induce functional changes in microglia toward an anti-inflammatory and neuroprotective function [43]. For example, butyrate treatment could suppress microgila activation and lipopolysaccharide (LPS)-induced depression-like behavior in mice [44]. However, SCFAs have been shown to promote amyloid β (Aβ) deposition in the brain of GF mouse model of Alzheimer’s disease (AD) by modulating microglia phenotypes [45]. Sex-specific effects of SCFAs on astrocyte gene expression have been investigated. For example, acetate upregulates the expression of the genes involved in anti-inflammatory pathways and propionate increases the expression of interleukin (IL)-22 in male, but not female cortical astrocytes [46]. Males are less likely to develop neuroinflammatory disorders than females, which reflects a possible neuroprotective pathway in males [47]. Apart from affecting microglia and astrocytes, SCFAs also directly influence neuronal development and function. SCFAs at physiologically relevant levels promote the proliferation and mitosis of human early neural progenitor cells [48]. Taken together, SCFAs play a role in the microbiota–gut–brain axis via modulation of the BBB or crossing of the BBB to further influence local cells in the CNS.

#### Tryptophan metabolites

The gut microbiota produce a diverse range of tryptophan metabolites, such as tryptamine and indoles. These metabolites can then signal locally to the intestinal mucosa and distant organs [49]. Researchers have recently investigated the potential role of tryptophan metabolites, which are produced by the gut microbiota, in the modulation of brain function [50, 51]. The gut microbiota that produce tryptophanases, such as *Lactobacillus* sp., *Escherichia coli*, *Clostridium* sp., and *Bacteroides* sp., can catalyze tryptophan to indoles and indole derivatives that can bind to their receptors, named aryl hydrocarbon receptor (AHR) [52, 53]. In addition, an in silico analysis indicated enrichment of tryptophan metabolism in five bacterial genera, including *Clostridium*, *Burkholderia*, *Streptomyces*, *Pseudomonas*, and *Bacillus*, suggesting that these bacterial groups have the ability to metabolize tryptophan in the gut [49]. Tryptophan metabolites produced by the gut microbiota regulate microglial activation as well as transforming growth factor alpha (TGFα) and vascular endothelial growth factor B (VEGF-B) production, and this may modulate the transcriptional program of astrocytes and limit CNS inflammation [54]. Indole is the major metabolite produced by the gut microbiota from tryptophan; it has been reported to impact neurogenesis and brain function [50, 51]. Indole derivatives can interact with AHR, which is normally expressed in the GI tract and in CNS cells, including neurons, astrocytes, and microglia [55]. Indole supplementation rescued adult neurogenesis in GF mice via AHR signaling in neural progenitor cells [50]. Administration of indole in rats resulted in a dramatic decrease in motor activity, and GF rats colonized by indole-producing bacterial species demonstrated enhanced anxiety-like behavior. This suggests that indole may play a critical role in promoting the development of anxiety and mood disorders [51]. In addition, indole derivatives, such as tryptamine, indole-3-acetic acid (IAA), and indole-3-propionic acid, can cross the BBB and regulate neuronal proliferation, differentiation, and survival through AHR signaling. Moreover, an in silico analysis showed that various tryptophan metabolites mediate the microbiota–gut–brain axis [49]. These studies suggest that tryptophan metabolites play an important role in the CNS.

#### Microbial neurotransmitters

Many neurotransmitters can be generated by the gut microbiota, such as γ-aminobutyric acid (GABA), catecholamines (dopamine and norepinephrine), and serotonin [56–58]. Lactic acid bacteria (LAB) can produce GABA from GABA-enriched fermented foods and beverages. LAB, such as the bacteria in the genera *Lactobacillus*, *Bifidobacterium*, and *Streptococcus*, produce the enzyme glutamic acid decarboxylase, which is used for GABA production [57, 59]. Among 91 culturable bacteria present in the human intestine, *Lactobacillus brevis* and *Bifidobacterium dentium* were found to be the most efficient GABA-producing bacteria [57]. GABA is the predominant inhibitory neurotransmitter in the CNS and exerts its inhibitory role in the immune system through two types of specific receptors, GABA_A_ and GABA_B_. Several studies showed evidence of the transport of GABA across the BBB, such as simple diffusion, the passing of solutes by transcytosis, or carrier-mediated transport, which probably allow small amounts of GABA to cross the BBB [60–62]. Using conditionally immortalized mouse brain capillary endothelial cell line as an in vitro BBB model, GABA transporter 2 (GAT2) and beta/GABA transporter-1 (BGT-1) expressed on the endothelial cells were found responsible for the GABA efflux transport across the BBB [60]. A study of radiolabelled GABA entering the brain in nenonatal and adult rat showed non-specific diffusion across the BBB mediated GABA transport [61]. Moreover, the evidence showed that nitric oxide may increase the BBB permeability, resulting in increased GABA entry into the brain [62]. GABA plays a role in the modulation of the inhibitory–excitatory balance necessary for brain function, down-regulation of cytokine release by proinflammatory immune cells, and secretion of neuropeptides by intrinsic and extrinsic intestinal nerve fibers [63–65]. GABA treatment was effective in ameliorating multiple sclerosis (MS) in an animal model and experimental autoimmune encephalomyelitis (EAE) by inhibiting inflammation [66].

Catecholamines, such as dopamine and norepinephrine, regulate several central and peripheral nervous system functions, including cognitive abilities, mood, and gut motility [67]. In the GI tract, dopamine and noradrenaline are mainly present in the colonic lumen. Levels of catecholamines have been found to be lower in GF mice than in specific pathogen-free mice; however, a mixture of *Clostridia* may elevate catecholamine levels in GF mice, suggesting that the gut microbiota plays a role in the generation of catecholamines in the gut lumen [68]. Beyond the gut, GF mice have also been found to have an increased turnover rate of dopamine and norepinephrine in the brain [16]. However, it is known that catecholamines are generally unable to penetrate the BBB, except at circumventricular sites, where the BBB is deficient or damaged [69]. For example, ethanol may facilitate the entry of catecholamines into the brain by enhancing the BBB permeability to catecholamines in chicks [70]. The influence of catecholamines on neurological disorders can occur in the CNS or in the peripheral tissues, which may further alter neurological function [71, 72]. Dopamine is a critical neurotransmitter that regulates peripheral immune responses and has been associated with several autoimmune diseases and neurological disorders [73, 74]. In the human, more than 50% of dopamine is synthesized in the gut, and peripheral dopamine levels can be regulated by the gut microbiota. A previous study showed that certain bacterial species in the genus *Staphylococcus* can produce dopamine via staphylococcal aromatic amino acid decarboxylase [75]. In addition, dopamine has also been found in the biomass of *Bacillus cereus*, *Bacillus mycoides*, *Bacillus subtilis*, *Proteus vulgaris*, *Serratia marcescens*, *Staphylococcus aureus*, and *Escherichia coli* [76]. Dopamine modulates the function of effector immune cells and the production of cytokines by activated T cells [77]. In the CNS, dopamine regulates nitric oxide synthesis and microglial cell migration [78, 79]. In addition, dopamine reduces the suppressive activity and migratory activity of Treg, which is implicated in neurodegeneration [71]. Calabresi et al*.* reported the role of dopamine in brain function; for example, the modulation of behavior, cognition, movement, emotions, memory, and learning [72]. Dysfunction of the dopaminergic system and altered immune function are associated with Parkinson’s disease (PD) [77]. Norepinephrine plays a role in sensory signal detection, working memory, behavior, and cognition. Reduced norepinephrine levels have been associated with depression, anxiety, and post-traumatic stress disorder [80]. Norepinephrine has been found in the biomass of the gut microbiota, including *Escherichia coli*, *Bacillus subtilis*, *Bacillus mycoides*, *Proteus vulgaris*, and *Serratia marcescens*, suggesting that these species might be able to produce norepinephrine [81]. In the brain, norepinephrine has neuroprotective effects by suppressing inflammatory gene transcription and enhancing BDNF production by microglia and astrocytes, which can further promote neuronal survival [82, 83]. In addition, norepinephrine can modulate excitatory and inter-neuronal responses [83].

Serotonin is another important neurotransmitter that carries signals between neurons throughout the body. GF mice showed a reduction in serotonin levels in the blood and colon [84] as well as an increased serotonin turnover rate in the brain [16]. Depleted serotonin levels may be restored via recolonization with several strains of bacteria, such as a consortium of spore-forming species. In addition, certain bacterial genera, such as *Candida*, *Streptococcus*, *Escherichia*, *Enterococcus*, and *Pseudomonas,* can produce serotonin [85]. In mammals, gut microbiota-derived serotonin can act locally in the intestinal tract or enter the blood circulation, but it does not cross the BBB. However, serotonin has been reported to increase BBB permeability, which indirectly impacts brain function [86]. Serotonin modulates several immune cell functions and is a known potent immune cell modulator in autoimmune diseases, via several mechanisms. For example, serotonin was shown to suppress MHC class II expression and the antigen-presenting capacity of macrophages [87]. Serotonin may also decrease the production of pro-inflammatory cytokines, such as IL-6 and tumor necrosis factor (TNF)-α, by macrophages and lymphocytes [88].

### Crosstalk with intestinal eptithelial cells (IECs)

The mucosal barrier is crucial for the maintenance of homeostasis of the body as it protects host tissues from environmental toxins and infections. The intestinal barrier plays an important role in preventing unwanted or harmful molecules from entering the body. The IECs secrete mucus, defensins, secretory–immunoglobulin A, and other mediators into the lumen, as well as produce essential mediators, such as peptides and neurotransmitters that are secreted into the lamina propria. The intestinal barrier is not only a physical barrier, but also regulates the absorption of dietary nutrients and water. The intestinal epithelium contains several intestinal stem cells (ISCs) and specialized IECs, such as absorptive enterocytes (the main cell population in the intestinal barrier), goblet cells, Paneth cells, and enteroendocrine cells. These specific IEC lineages form a gut barrier and play different roles in maintaining host homeostasis. Therefore, alteration of the intestinal barrier or specific IEC lineages may impact the host status and lead to diseases in local and distant tissues, such as gastrointestinal and neurological disorders. In this section, we discuss how IECs mediate the communication between the gut microbiota and the brain.

#### The intestinal epithelium and enterocytes

The intestinal epithelium is composed of a single continuous layer of specialized IECs that function as an intestinal barrier and separate the internal milieu from the intestinal lumen. To maintain homeostasis, the intestinal epithelium limits contact between the host and the massive load of luminal molecules. The molecules in the intestinal lumen can pass through the intestinal barrier via two routes: paracellular passage and transcellular passage. The paracellular passage is a pathway that allows small molecules to diffuse through the TJs between adjacent IECs. The transcellular passage is a pathway that enables the transfer of larger molecules through endocytosis or exocytosis of IECs [89]. The main IECs that populate the intestinal epithelium are enterocytes. Besides their absorption function, enterocytes also act as non-professional antigen-presenting cells (APCs) and release cytokines in response to stimuli [90]. Enterocytes are tightly connected to one another through the apical junctional complex composed of TJs, adherence junctions, and desmosomes. The proteins in this complex provide the strength to hold the cells together and regulate intestinal permeability. Leaky gut is a condition characterized by the loss of intestinal barrier integrity and increased intestinal permeability, resulting in uncontrolled translocation of bacteria and harmful substances into the lamina propria and bloodstream and sequentially inducing inflammatory responses Therefore, leaky gut has been implicated in various diseases, including neurological diseases, such as AD, PD, chronic depression, autism spectrum disorder (ASD), and MS [91]. The intestinal barrier is disrupted in patients with PD and PD mouse models, suggesting that intestinal barrier–brain interaction plays an important role in PD pathology [92]. Increased intestinal permeability characterized by increased translocation of LPS from gram-negative enterobacteria promotes the inflammatory pathophysiology of depression [93]. The gut microbiota composition and bacterial metabolites are involved in the regulation of the intestinal barrier integrity and the CNS consequences [94, 95]. SCFA-producing bacteria, SCFAs, and tryptophan metabolites have been shown to regulate intestinal permeability [95]. SCFAs can enhance the intestinal barrier by upregulating TJ protein expression and facilitating TJ assembly [96, 97]. The modulation of TJs in the intestinal barrier by the gut microbiota and bacterial metabolites may also regulate BBB permeability, because there are several similar TJ proteins between the intestinal barrier and the BBB [22, 98].

#### Goblet cells and mucus production

Goblet cells secrete mucin, which is glycosylated and polymerized into a net-like structure called the mucus layer. The mucus layer is defined as two connected layers: an outer loose mucus layer and an inner adherent mucus layer [99]. The presence of a very high number of bacteria provides an impenetrable mucus layer. The composition of the microbiota can regulate mucus layer properties, influencing its permeability [100]. The mucus layer also serves as an energy source, mainly in the form of glycans, for the gut microbiota residing in the mucus layer. Many gut microbiota are known to be mucin-degrading microorganisms, such as *Akkermansia muciniphila* [28] and *Bacteroides thetaiotaomicron* [101], and are implicated in increasing the numbers of goblet cells and stimulating mucin production [102]. The products of mucus degradation can be utilized by other gut microbiota, such as *Lachnospiraceae* [103], *Clostridium cluster XIV* [104], *Clostridium difficile* [105], and *Enterobacteriaceae* [106]. GF animals showed fewer and smaller goblet cells as well as a relatively thinner mucus layer than conventional mice, suggesting that the gut microbiota plays a role in mucus composition and thickness [107, 108]. It has been shown that the thickness of the mucus layer may be restored when GF mice are exposed to bacterial products (peptidoglycan or LPS) [109]. In addition, certain gut microbiota, such as *Lactobacillus plantarum* and *Lactobacillus rhamnosus* GG, may induce the expression of mucins [110]. Abnormal mucin production and altered mucus layers are associated with neurological disorders, such as Alzheimer’s disease, PD, and MS [111]. A recent study demonstrated that the administration of umbilical cord mesenchymal stem cells through intranasal instillation corrected the microbial composition, maintained intestinal goblet cells, and improved locomotor function in PD, suggesting a positive correlation between goblet cells and PD [112]. *A. muciniphila* has been reported to stimulate mucus synthesis and mucus degradation. It may be hypothesized that mucus degradation may lead to a compensatory increased systhesis of mucus [102]. Treatment with *A. muciniphila* alleviated the reduction of colonic mucus cells and relieved cognitive impairment and anxiety-related behaviors in an AD mouse model [113]. These finding suggest that the reduction of goblet cells is associated with neurological disorders.

#### Enteroendocrine cells (EECs) and peptide/hormone production

The EECs are distributed along the entire GI tract and comprise approximately 1% of the overall IEC population. The diversity of EEC populations is generally lower in the colon than in the small intestine [114]. The EECs play an important role in the GI tract, such as GI secretion and motility, food intake regulation, and gut hormone production. After sensing stimuli in the luminal content, EECs produce and release signaling molecules or hormones that can act locally on neighboring cells in the GI tract or enter the blood circulation to act on distant target tissues [114, 115]. Various types of EECs can be identified by the hormones they produce. For instance, secretin-secreting S cells, motilin-secreting M cells, and neurotensin-secreting N cells are only present in the small intestine [116]. Three main types of EECs prevalent within the lower GI tract have been described, including enterochromaffin (EC) cells, D-cells, and L-cells [117]. Many peptides or hormones are produced by these EECs, such as serotonin (5-HT), PYY, GLP-1, GLP-2, and somatostatin. EC cells are the most abundant EECs and are widely distributed throughout the gastrointestinal tract. EC cells mainly produce 5-HT, which acts on its receptors that are expressed on various cell types, including enteric neurons, EC cells, and absorptive enterocytes. The gut microbiota and their bacterial metabolites can promote the differentiation of ISCs toward the secretory 5-HT-producing lineage and stimulate 5-HT secretion, resulting in increased circulating 5-HT levels [118, 119]. In addition, metabolites from spore-forming bacteria upregulate the expression of the tryptophan hydrolase 1 gene, resulting in increasing biosynthesis of serotonin by ECs [120]. Serotonin is hardly able to cross the BBB. The bioavailability of serotonin in serum is linked to a number of neurological disorders. For example, serum levels of serotonin have been found to be lower in patients with AD [121] and PD [122]. However, the evidence showing the indirect effect of serotonin on the CNS and how an alteration in serotonin leads to neurological disorders still need to be elucidated. L-cells constitute the second largest population of EECs and are found throughout the small intestine and colon. The dominant secretory products from L-cells are GLPs and PYY. L-cells sense bacterial metabolites and secrete GLP-1 and PYY locally into the blood circulation [36, 123]. Both GLP-1 and PYY can cross the BBB and interact with their receptors expressed on nerve cells, resulting in neuroprotective effects [124–126]. Apart from having a role in the treatment of type 2 diabetes mellitus, GLP-1 plays a beneficial role in MS, AD, PD, and hypertension [127, 128]. Evidence showed that systemically infused labeled GLP-1 crossed the BBB through active trans-endothelial transport which requires GLP-1 receptor (GLP-1R) binding [125]. GLP-1R activation stimulates neuronal proliferation and neural stem cell differentiation. GLP-1R stimulation also improves neuronal disorder features, such as memory dysfunction, neuromotor impairment, and neuronal degeneration [129]. In addition, GLP-1 has recently been shown to regulate neuroinflammation, neurogenesis, and synaptic function in the alleviation of depression [130]. PYY can react with a neuropeptide Y receptor on neurons, resulting in the inhibition of food intake. The augmentation of neuropeptide Y receptor by PYY leads to alterations in social interaction, sensorimotor function, learning, and memory [131]. Moreover, recent evidence showed that altered PYY and its receptor signaling may play an important role in anxiety-related and depression-like behaviors [132, 133].

### Immune system

The immune system serves as an important coordinator of the gut microbiota–brain axis. The gut microbiota not only modulate gut-resident immune cells but also brain-resident immune cells [134]. Activation of the immune system in both the gut and brain is implicated in the response to neuroinflammation, which further contributes to the pathology of neurological disorders. Microbe-associated molecular patterns (MAMPs) are normally recognized by toll-like receptors (TLRs) that are expressed on various types of immune cells, resulting in immune cell activation. Activated immune cells produce pro-inflammatory cytokines, such as IL-1β, IL-17A, TNF-α, and IL-6, which enter the brain circulation through the BBB, and may result in the development and progression of several neurological disorders [135]. In the EAE model, GF mice produce lower levels of pro-inflammatory cytokines, interferon (IFN)-γ and IL-17A, in the intestine and spinal cord. In addition, the colonization of segmented filamentous bacteria (SFB) induces Th1 and Th17 responses in the intestine and spinal cord and promotes EAE symptoms in GF mice [136]. Conversely, *Bacteroides fragilis* and *Prevotella histicola* colonization can suppress EAE by promoting Treg function, suggesting that the gut microbiota modulate neuroinflammation via immune responses [137]. SFB colonization is also sufficient to promote ASD-like symptoms through the modulation of Th17 cells in the intestine; however, blocking IL-17A by neutralizing antibodies can limit the behavioral abnormalities related to ASD [138]. Inflammasome activation causes the maturation of caspase-1 and the release of pro-inflammatory cytokines IL-1β and IL-18, which are involved in neuroinflammation. Specific MAMPs can activate inflammasome pathways and pro-inflammatory cytokine production, which have been implicated in a wide range of neurological disorders [139]. Moreover, mice with a genetic deficiency of caspase-1 have decreased depressive- and anxiety-like behaviors following chronic stress [140].

In addition, the gut microbiota also has a direct effect on CNS-resident immune cell function. In the brain, gut microbiota-derived molecules that can cross the BBB may affect the maturation and activation of brain immune cells, such as microglia and astrocytes [134]. Microglia in the CNS contribute to brain development, homeostasis, and pathology. Like other tissue-resident macrophages, microglia exert their functions in the CNS through cytokine release, complement activation, and phagocytosis [141]. Microglia in the gut microbiota-depleted mice showed altered inflammatory gene expression profiles and an immature state [20]. However, the mechanism by which the gut microbiota can influence microglia remains unclear. Besides microglia, astrocytes are major immune cells among glial cells that participate in several functions, including control of the BBB, regulation of CNS development and repair via the production of cytokines and chemokines, as well as antigen presentation. Type 1 IFN signaling in astrocytes, mediated by microbial tryptophan metabolites and AHR activation, can limit CNS inflammation [52, 142]. Regulation of immune cell homeostasis could be an alternative strategy to control communication in the gut microbiota–brain axis.

## Alteration of the gut microbiota and immune-related neurological disorders

Bidirectional communication in the gut microbiota–brain axis reveals a complex process that ensures the maintenance of both gastrointestinal and brain homeostasis. Many studies have shown that microbiome–immune crosstalk contributes to neurological disorders, such as developmental disorders, neurodegeneration, and emotional dysregulation, which will be reviewed in this section.

### Developmental disorders

#### Autism spectrum disorder

ASD is a group of neurodevelopmental disorders characterized by deficits in social communication and behaviors. Patients with ASD also have GI disturbances, such as barrier disruption, constipation, abdominal pain, and diarrhea, which are linked to the severity of ASD symptoms [143]. Increased intestinal permeability has been correlated with behavioral severity in very young children with ASD [144]. Elevated serum levels of toxins and bacterial products, a result of increased intestinal permeability, can induce immune responses related to impaired brain function and social behavior [145, 146]. Human gut microbiota from ASD can promote ASD behavior in mice. Specific bacterial taxa and their metabolites were predicted to modulate ASD behavior of mice harboring human microbiota. Furthermore, treatment with the microbial metabolites depleted in ASD improved behavior in mice, pointing to the involvement of the gut microbiota and bacterial metabolites in ASD [4]. Recent studies have revealed that ASD is often associated with altered gut microbiota composition and dysregulated immune responses [146–149]. Low levels of the genera *Prevotella*, *Coprococcus*, and unclassified *Veillonellaceae* were observed in children with ASD and GI disorders [150]. A meta-analysis has also revealed that children diagnosed with ASD had lower levels of *Enterococcus*, *Escherichia coli*, *Bifidobacterium*, and *Bacteroides* populations and higher levels of *Faecalibacterium*, *Lactobacillus,* and *Ruminococcus* populations [148]. In fecal samples of children with ASD, researchers found increased levels of the *Clostridium histolyticum* group (*Clostridium* clusters II and I), which are recognized as producers of toxins [149]. The reduction of the levels of these *Clostridia* by vancomycin treatment has been shown to improve ASD features [151], suggesting that bacteria in the *C. histolyticum* group may contribute to ASD-like symptoms. Besides GI disturbances and alteration of the gut microbiome, immune dysfunction and autoimmunity are highlighted as key players contributing to the pathogenesis of ASD [152].

Many studies demonstrated that an alteration of the gut microbiome during pregnancy leads to ASD in offspring and this effect is mediated by immune response modulation [138, 153]. A murine maternal immune activation (MIA) model is used to study neurodevelopmental disorders, such as ASD. Choi et al*.* showed that Th17 cells and the effector cytokine IL-17A are key factors in mothers for MIA-induced ASD in offspring [153]. This study revealed that maternal IL-17A and fetal brain IL-17 receptor levels were elevated in MIA and maternal IL-17A promotes abnormal brain development and ASD-like behavioral phenotypes in offspring. However, these effects could be rescued by treatment with anti-IL-17A antibody during pregnancy. Lammert et al*.* demonstrated that the prenatal gut microbiota composition influenced the development of ASD-like phenotypes through the modulation of maternal IL-17A signaling in the MIA model [138]. These studies suggest that the modulation of maternal gut microbiota composition and the inhibition of IL-17A signaling may represent a good strategy for protection against ASD. Hsiao et al*.* revealed an alteration of the gut microbiota and GI barrier defects in MIA [146]. However, the altered microbial composition, gut permeability, and ASD-related defects were improved by treatment with the human commensal *Bacteroides fragilis* in MIA offspring. In addition, naïve mice treated with a metabolite that is increased by MIA caused ASD-related behavioral abnormalities, supporting the microbiota–gut–brain axis in ASD [146]. Mice transplanted with human ASD microbiome exhibits ASD-like behaviors, and the administration of the microbial metabolites depleted in ASD has been shown to improve behavioral abnormalities, suggesting that the microbiome contributes to ASD symptoms via the production of neuroactive metabolites [4]. In addition to the gut microbiota alterations, changes in microbial metabolites have also been noted in ASD [154, 155]. Kang et al*.* showed that isopropanol concentrations were higher and GABA concentrations were lower in the feces of children with ASD [154]. Levels of SCFAs, including acetic acid, propionic acid, butyric acid, isobutyric acid, valeric acid, isovaleric acid, and caproic acid, were elevated in fecal samples of children with ASD [155]. The effects of propionic acid on the pathogenesis of ASD have also been demonstrated [156, 157]. Intraventricular administration of propionic acid induced abnormal movements, cognitive deficits, and impaired social interactions in rats. Moreover, increased oxidative stress and neuroinflammation have been observed in the brain tissue of propionic acid-treated rats [156]. An in vitro study using human neural stem cells showed that propionic acid induced glial cell differentiation, gliosis, and pro-inflammatory cytokine release [157].

Various factors related to the gut microbiota communities may impact ASD, including prenatal maternal factors, such as health condition [158, 159], the use of antibiotics [160], mode of delivery [161, 162], and feeding patterns [163–165]. A study of medical records showed that maternal obesity was positively associated with having a child with ASD [158]. In a mouse model, feeding a mother on a high-fat diet induced dysbiosis of the gut microbiota and social behavior deficits in the offspring [159]. In addition, the use of various antibiotics by mothers during pregnancy has been shown to cause deficiencies in fetal neurodevelopment and ASD [160]. This association might be due to an alteration in the maternal gut microbiota and immune activation by antibiotics [166, 167]. However, antibiotic use during the first year of life in offspring was not associated with the development of ASD [168]. Studies on the etiological relationship between cesarean section (c-section) and ASD showed that infants delivered by c-section showed a high probability of developing ASD [161, 162]. In an ASD mouse model, c-section delivery caused social behavioral abnormalities in offspring. However, treatment with oxytocin, a known regulator of social behavior, recovered the low sociability of mice delivered via c-section [162]. Previous studies have reported that in children with earlier initiation of breastfeeding, increased feeding periods, and continued breastfeeding, the risk of ASD was decreased [163, 164]. Moreover, over 6 months of breastfeeding has been associated with lower rates of ASD development and ASD-related GI symptoms [165]. These findings suggest that gut microbiome and immune responses during pregnancy and early life factors can initiate ASD-like behaviors in offspring.

### Neurodegeneration

#### Parkinson’s disease

PD is a progressive, age-associated neurodegenerative disease characterized by the loss of dopaminergic neurons in the CNS, and, eventually, in the motor system. The key pathological characteristics of PD are the accumulation of the protein alpha-synuclein (α-syn) and cell death, especially of dopamine-secreting neurons in the brain. The association between autoimmune diseases and PD has been demonstrated, for example, impaired cellular and humoral immune responses as well as immune dysregulation in PD [169]. Pathway-based analysis showed that the genes involved in the regulation of leukocyte/lymphocyte activity and cytokine-mediated signaling are associated with the risk of PD [170]. Accumulating evidence suggests that intestinal inflammatory responses and intestinal derived inflammation related to dysbiosis play pathological roles in PD [169, 171]. Most patients with PD have increased intestinal permeability, which may reflect gut microbiota disorders [172], and this is reported to facilitate motor deficits, microglial activation, and α-syn pathology [173]. A mouse model of PD demonstrated that the gut microbiota regulated pathways that induce α-syn aggregation and prevent the clearance of insoluble protein aggregates. In addition, the gut microbiota promoted α-syn-dependent microglial activation and motor dysfunction [173]. Therefore, immune-based therapeutic strategies for PD have been developed, for example, immunotherapy targeting α-syn and immune mediators [169]. Excessive bacterial growth in the small intestine was found in patients with PD; it is related to abnormal absorption and motor impairment [174]. Mice harboring the gut microbiota derived from patients with PD showed increased motor impairment [173]. Compared to healthy controls, patients with PD exhibit lower levels of *Bacteroides*, *Lactobacillus*, *Prevotella*, *Peptostreptococcus*, and *Butyricoccus* spp., and higher levels of *Lactobacillus*, *Enterobacter,* and *Proteus* spp. [173, 175]. Analysis of mucosal and fecal microbial communities of patients with PD versus healthy subjects showed that the levels of butyrate-producing bacteria from the genus *Blautia*, *Coprococcus*, and *Roseburia* were higher in feces of healthy participants, and bacteria from the genus *Faecalibacterium* were more abundant in the mucosa of healthy participants than in that of PD patients [176]. Moreover, different stages of the disease display different gut microbiota alterations. The level of *Clostridium coccidias* was found to be increased in early PD, whereas *Lactobacillus gassier* was increased in advanced PD [175]. Keshavarzian et al*.* found lower levels of butyrate-producing bacteria and a reduction in SCFA levels in patients with PD, which may eventually result in increased mucosal permeability and systemic endotoxin exposure from coliform bacteria [176]. Recent studies have also observed a reduction in the SCFA levels in participants with PD, which is consistent with alterations in the gut microbiota composition [177, 178]. Unger et al*.* showed that SCFA concentrations in fecal samples were decreased in patients with PD compared to age-matched controls [177]. Aho et al*.* demonstrated that SCFA concentrations were reduced in the stool of patients with PD in a sex-dependent manner, and that the gut microbiota diversity and composition were inversely associated with the levels of SCFAs [178]. The abundances of *Butyricicoccus*, *Clostridium*, and *Roseburia* were positively correlated with SCFAs levels; in contrast, the levels of *Akkermansia*, *Escherichia*/*Shigella*, *Flavonifractor*, *Sporobacter*, *Intestinimonas*, and *Phascolarctobacterium* were negatively correlated with the levels of SCFAs. In addition, researchers also found that stool SCFA levels were related to the onset and symptom severity of PD. Several studies have revealed the protective effects of butyrate in a PD mouse model [179, 180]. PD mice treated with sodium butyrate showed improvements in neurobehavioral impairment, prevented dopaminergic generation, attenuated the PD-associated disruption of BBB, and increased colonic GLP-1 and brain GLP-1R [179]. A drosophila model of PD showed that sodium butyrate-supplemented food could rescue local motor impairment, which was associated with elevated levels of dopamine in the brain [180]. However, the role of SCFAs in PD remains unclear. Several studies have demonstrated the negative effects of SCFAs in PD pathologies [173, 181]. For example, the administration of a mixture of SCFAs (acetate, propionate, and butyrate) in mice overexpressing α-syn under GF conditions induced neuroinflammation by promoting α-syn aggregation, microglial activation, and motor deficits [173]. Qiao et al*.* showed that sodium butyrate exacerbated the decline of dopaminergic neurons, aggravated neuroinflammation by increased microglial and astrocyte activation, and promoted colonic inflammation in an MPTP-treated mouse model of PD. In addition, an in vitro study using a BV2 mouse microglial cell line showed that sodium butyrate upregulated the expression of inflammatory mediators in LPS-stimulated BV2 cells [181].

#### Alzheimer’s disease

AD is a progressive neurodegenerative disorder characterized by the presence of extracellular aggregates of Aβ, tau pathology, neurofibrillary tangles, neuronal loss, and neuroinflammation [182–184]. It is widely known that Aβ plays a central role in AD initiation, while neuroinflammation influences the progression of cognitive decline. TLRs expressed by microglia recognize soluble Aβ peptides and induce inflammasome complexes, resulting in the initiation of neuroinflammatory responses [185]. In addition, peripheral immune cells, specifically type I interferon responses from T cells, also play a role in CNS neuroinflammatory responses [183]. The microtubule-binding protein tau is predominantly localized in the axons in mature neurons to stabilize the microtubule structure and neuronal connectivity [186]. In AD, misfolded and hyperphosphorylated tau proteins accumulate in neurons; this is associated with altered protein turnover at synapses [184, 187]. Of recent, many studies have proposed a potential role of gut microbiota alteration in the development or exacerbation of AD pathology [45, 188–191]. Antibiotic-induced microbiota alteration plays a key role in modulating neuroinflammation, which in turn has been shown to influence amyloidosis in an AD mouse model [188]. The transfer of a healthy gut microbiota could reduce aggregates of Aβ, tau pathology in the brain of AD mice [192]. Administration of *L. plantarum* could prevent cognitive dysfunction by suppresing Aβ plaque deposition and tau hyperphosphorylation in AD mice [193]. SCFAs derived from the gut microbiota contribute to the pathology of AD by increasing microglial activation and Aβ deposition [45]. The gut microbiota dysbiosis exacerbates the progression of Alzheimer’s disease in flies by recruiting hemocytes to the brain and causing neuroinflammation [191]. Patients with AD have an imbalance in the gut microbiota and decreased microbial diversity. In patients with AD, the levels of beneficial gut microbiota, such as *Eubacterium rectale*, *Bifidobacterium*, and *Dialister,* have been shown to be decreased, while the levels of pathogenic gut microbiota, including *Escherichia*/*Shigella*, *Bacteroides*, and *Ruminococcus*, are increased [189, 190]. In addition, there is a positive correlation between the levels of *Escherichia*/*Shigella* and pro-inflammatory cytokines IL-1β and CXCL2 in the serum of patients with AD, suggesting that alteration of these gut microbiota taxa is possibly associated with peripheral inflammation in patients with AD [189]. The contributions of the gut microbiota to peripheral and central immunological changes in AD are described [194]. Peripheral blood lymphocytes can enhance BBB permeability and infiltrate into the brain, resulting in the release of pro-inflammatory cytokines affecting Aβ production/deposition in the brain [195]. Therefore, regulation of the gut microbiota and immune responses, such as treatment with probiotics and prebiotics, is considered a therapeutic strategy for AD.

#### Multiple sclerosis

MS is one of the inflammatory autoimmune diseases characterized by the breakdown of the BBB and demyelination due to the infiltration of self-reactive T cells. EAE is an animal model of MS, characterized by an increase in proinflammatory cytokine-producing immune cells, such as Th1 and Th17. A recent study showed that GABA produced by *Lactobacillus brevis* exhibited inhibitory effects on the proliferation and production of IFN-γ and IL-17 by mesenteric lymph node cells, as well as the expression of costimulatory molecules on APCs. In contrast, GABA induces the expression of immunoregulatory molecules, including Foxp3^+^, IL-10, and TGF-β [196]. GABA may ameliorate EAE via inhibition of inflammation, directly acting on APCs and adaptive immune cells in response to myelin proteins [66]. Dopamine levels are lower, whereas the percentage of IL-17 and IFN-γ producing cells is higher in MS patients than in healthy subjects, suggesting a suppressive effect of dopamine in MS [73]. MAMPs are detected by TLRs expressed on various types of immune cells. For example, polysaccharide A produced by *Bacteroides fragilis* in the human gut microbiota is recognized by TLR2, which mediates the expansion of Treg, resulting in protection against CNS demyelination and inflammation in EAE [142]. In addition, the type I interferon and AHR axes activated by bacterial tryptophan can limit CNS inflammation [52]. Dopamine can downregulate the production of IFN-γ and IL-17 by peripheral blood mononuclear cells (PBMCs) in patients with relapsing–remitting MS, suggesting the potential role of dopamine in MS therapy [197]. Several studies have focused on the beneficial role of SCFA-producing bacteria and SCFA metabolites in MS therapy [198, 199]. Patients with MS have an altered gut microbiome with depletion of SCFA-producing bacteria and a significant reduction in SCFA concentrations [198, 200]. In a proof-of-concept study by Duscha et al., treatment with propionic acid for 2 weeks in patients with MS reduced Th1 and Th17, whereas Treg and Treg-inducing genes were increased. In addition, supplementation with propionic acid for 3 years reduced the annual MS relapse rate and brain atrophy [198]. Haghikia et al. showed that SCFAs exert anti-inflammatory effects on T cell proliferation and differentiation. Mice treated with propionic acid showed induction of Tregs in the small intestine, reduced lymphocyte infiltration, and demyelination in the EAE animal model [199]. A reduction in serotonin levels was found in patients with MS and EAE mice [201, 202]. Previous studies have shown that elevation of serotonin levels could cause immune-modulation effects and reduce the MS/EAE progression rate [203]. For example, increasing serotonin levels could attenuate disease severity by reducing T cell proliferation, suppressing the release of IL-17 and IFN-γ, and inducing IL-10 production [203]. In addition, serotonin may influence macrophage polarization into M2 macrophages in MS pathological processes [204]. Moreover, the activation of GLP-1/GLP-1R signaling in microglia improved clinical symptoms and reduced spinal cord damage in EAE mice [205]. Therefore, modulation of the gut microbiota, bacterial metabolites, and IEC-derived neuropeptides may serve as therapeutic strategies for EAE.

### Mood and emotional effect

#### Depression and anxiety

Depression is a psychiatric disorder. Various socioeconomic factors and sex may influence the rate of depression [206]. Anxiety is characterized by feelings of unease and nervousness. Mice subjected to chronic restraint stress were found to have altered compositions of the gut microbiota compared to control mice [140, 207]. Wong et al. demonstrated that the relative levels of bacteria in the genera *Allobaculum*, *Bifidobacteria*, *Turicibacter*, *and Clostridium* were reduced, and the relative level of the family *Lachnospiraceae* was increased in mice with chronic restraint stress [140]. Wu et al*.* identified 29 differentially abundant bacterial taxa between depressed mice and control mice, especially bacteria in the genus *Allobaculum* and family *Ruminococcaceae *[207]. Moreover, the researchers showed that the levels of acetic acid, propionic acid, pentanoic acid, norepinephrine, and serotonin were decreased in depressed mice. In addition, the levels of bacteria in the genus *Allobaculum* were positively correlated with acetic acid and serotonin levels, suggesting that alterations of the gut microbiota and its metabolites or neurotransmitters may influence depression. Simpson et al*.* reviewed gut microbiota alterations associated with depression and anxiety [208] and found that higher levels of proinflammatory bacteria, such as *Enterobacteriaceae* and *Desulfovibrio*, and lower levels of SCFA-producing bacteria, such as *Faecalibacterium*, may be related to the pathophysiology of depression and anxiety. The GABAergic system is important for protection against the development of depression and anxiety disorders. Treatment with *Lactobacillus rhamnosus* JB-1 in mice reduced depressive and anxiety-like behaviors by changing the cerebral GABAergic activity [209].

## Conclusions

It is now being accepted that alterations in the gut microbiota or disruptions in the microbiota–gut–brain axis may directly or indirectly impact brain function. Through bacterial metabolites/neurotransmitters, IECs, and the immune system, the gut microbiota seems to contribute to the regulation of neurophysiological function and cognition. The link between the microbiota–gut–brain axis and immune-related neurological disorders is gaining the attention of late. Various strategies have been used to investigate the role of the microbiota–gut–brain axis in immune-related neurological disorders, including GF studies, infection studies, probiotic studies, antibiotic studies, and fecal transplantation studies. Although studies of the microbiota–gut–brain axis have flourished in recent years, the methods of clarifying the direct effects of the gut microbiota on the brain are limited. For example, there is a need to include the complications of the BBB in the study of bidirectional communication between the gut microbiota and the brain. To move beyond correlative studies, new advanced technologies are being developed to discover and validate biological mechanisms of action and to develop treatments for neurological diseases. A deep understanding of the microbiota–gut–brain axis may aid the development of treatments that can improve the brain function of individuals with neurological diseases. The gut microbiota-based therapy may serve as a promising approach in the treatment of neurological disorders in the future.

## Data Availability

Not applicable.

## Abbreviations

α-syn: Alpha-synuclein
5-HT: 5-Hydroxytryptamine
Aβ: Amyloid β
AD: Alzheimer’s disease
AHR: Aryl hydrocarbon receptor
APC: Antigen-presenting cell
ASD: Autism spectrum disorder
BBB: Blood–brain barrier
BDNF: Brain-derived neurotropic factor
c-section: Cesarean section
CNS: Central nervous system
EAE: Experimental autoimmune encephalomyelitis
EC: Enterochromaffin
EEC: Enteroendocrine cell
GABA: γ-aminobutyric acid
GF: Germ-free
GI: Gastrointestinal
GLP-1R: Glucagon-like peptide-1 receptor
GLP: Glucagon-like peptide
GPR: G protein-coupled receptor
IAA: Indole-3-acetic acid
IEC: Intestinal epithelial cell
IL: Interleukin
ISC: Intestinal stem cell
LAB: Lactic acid bacteria
LPS: Lipopolysaccharide
MAMP: Microbe-associated molecular pattern
MCT: Monocarboxylate transporter
MIA: Maternal immune activation
MS: Multiple sclerosis
PBMC: Peripheral blood mononuclear cell
PD: Parkinson’s disease
PYY: Peptide YY
SCFA: Short-chain fatty acid
SFB: Segmented filamentous bacteria
TJ: Tight junction
TLR: Toll-like receptor
TNF: Tumor necrosis factor
Treg: Regulatory T cell
